# On the Treatment of Tetanus

**Published:** 1861

**Authors:** 


*On the Treatment of Tetanus.*—By Editor of 'Lancet.'—Of the different indications in the treatment of tetanus, besides the removal of any source of irritation, and the support of the general strength, but especially of the heart, two are prominently dwelt upon by writers, namely, to lessen the susceptibility of the nervous centres to any irritating influence which may exist, and to diminish the irritation by means calculated to depress nervous excitement. To accomplish the first, Dr. Wood, of Philadelphia, thinks the weight of testimony is greatly in favor of opium, notwithstanding the contrary judgment of some distinguished authors. It has probably been employed, he asserts, as one of the remedies in the great majority of cases. It has failed like other remedial agents, but in many cases all prove ineffective. The liquid forms, from their more ready absorption, are to be preferred to the solid. The second indication is fulfilled by measures that have a sedative influence on the nervous system, and by those which act revulsively. Into these we do not propose to enter, but we may state that the tobacco enema (half a drachm to the half-pint of boiling water) is highly praised by Dr. Wood. It is to be repeated in an hour, and then every two or three hours, till the relaxing effects are produced. The nervous system is easily impressed by the influence of tobacco, as contrasted with opium; and we think that by-and-by, when the question has been thoroughly tested, it will be found that tobacco or its alkaloid, nicotine, is of greater value than many other remedies in tetanus. The revulsive plan consists in blisters or caustic applications to the whole length of the spine; or ice may be advantageously employed. The latter remedy is asserted to have been the means of cure in sixteen out of seventeen cases under the care of Dr. Carpenter, of Suffolk county, New York.—*Lancet*, *Dec.* 6, 1860, *p.* 560.

---

THE LAMP BATH.—The following is a very simple and most effectual method of exciting the functions of the skin. Let the patient, in *puris naturalibus*, be seated on a common wooden chair with his feet upon a low stool the body then enveloped in two or three blankets, the head being excluded, and a large spirit-lamp placed under the seat. In about a quarter of an hour, the perspiration streams down the skin.—After a time the blankets must be removed, and the patient subjected to a douche of two pailsful of cold water, and then dried with much friction. After which a smart walk may be taken. (Dr. C. Taylor, p. 282.)

